# Associations of viral infections and antiviral vaccinations with anti-NMDAR and other forms of autoimmune encephalitis

**DOI:** 10.1016/j.bbih.2026.101301

**Published:** 2026-07-06

**Authors:** Sarah Bamberg, Poul M. Schulte-Frankenfeld, Jakob Kreye

**Affiliations:** a Charité – Universitätsmedizin Berlin, Freie Universität Berlin and Humboldt-Universität zu Berlin, Department of Pediatric Neurology, Berlin, Germany; b Berlin Institute of Health at Charité – Universitätsmedizin Berlin, Germany

**Keywords:** Viral trigger, Herpes simplex virus, Anti-NMDAR encephalitis, Post-infectious autoimmunity, Molecular mimicry, Autoantibodies

## Abstract

**Background:**

Autoimmune encephalitis (AIE) comprises a diverse group of neurological disorders characterized by autoantibodies targeting specific antigens of the central nervous system (CNS). While the pathophysiological effects of these autoantibodies have been extensively studied, the principles and immunological triggers underlying their generation remain less well understood. Viral infections and antiviral vaccinations have been reported as potential immunological triggers. However, a systematic overview of their specific associations and frequencies across AIE subtypes is lacking.

**Methods:**

Here, we performed a systematic literature review in PubMed to identify published cases of AIE linked to viral infections or antiviral vaccinations, and to test whether herpes simplex virus (HSV)-associated anti-N-methyl-D-aspartate receptor encephalitis (NMDAR-E) represents a disproportionately strong association. We quantified the frequencies of viral infection- or vaccine-related AIE across ten common antibody-defined subtypes, and characterized their epidemiological and clinical features, complemented by data from 23 population-based viral encephalitis cohorts.

**Results:**

We identified 556 cases, of which 488 were infection-associated and 68 followed vaccination. The most frequent combination was HSV infection in association with NMDAR-E, which outnumbered all other reported virus-AIE associations combined and was almost exclusively attributable to preceding HSV encephalitis (HSE). Other recurrent associations included Japanese encephalitis virus, Epstein-Barr virus (EBV), and SARS-CoV-2 infections, although EBV- and SARS-CoV-2-associated cases were less clearly suggestive of causal post-infectious autoimmunity. Across 23 population-based viral encephalitis cohorts from five continents, HSV was the leading causative pathogen. Despite this high background frequency, HSV was significantly overrepresented among viral CNS infection-associated NMDAR-E cases in our dataset compared with the reference cohorts (*P* < 0.0001), indicating a disproportionate association. Most reported HSE-associated NMDAR-E cases originated from Europe and occurred in young children (median age 6 years), with a balanced sex distribution and a median interval between infection and NMDAR-E onset of 30 days.

**Conclusion:**

Collectively, these findings provide the most comprehensive overview of viral infection- and antiviral vaccination-associated AIE to date and support a pathogen-specific link between HSE and secondary NMDAR-E as the most dominant virus-AIE association in the published literature. They underscore the need to elucidate the underlying immunological mechanisms and to develop biomarkers predictive of secondary NMDAR-E.

## 1 Introduction

Autoimmune encephalitis (AIE) describes a group of inflammatory neurological disorders, each characterized by autoantibodies against a distinct antigenic target within the central nervous system (CNS) (Dalmau and Graus, 2018). Patients typically present with rapidly progressive neuropsychiatric symptoms that often respond to timely and sufficiently intensive immunosuppressive therapies (Pruss, 2021; Titulaer et al., 2013). Clinical outcome strongly depends on early diagnostic confirmation and prompt treatment initiation (Nosadini et al., 2021; Titulaer et al., 2013), underscoring the importance of a detailed understanding of early events and risk factors underlying subtype-specific disease pathogenesis. In comparison, the CNS demyelinating disease multiple sclerosis (MS) has long been suspected to have a viral trigger. This link was recently substantiated by a large cohort study of more than 10 million young adults demonstrating that Epstein-Barr virus (EBV) infection is a prerequisite for MS onset (Bjornevik et al., 2022). This epidemiological confirmation of a distinct viral association has provided novel insights into MS pathophysiology and has directly informed diagnostic biomarker discovery and the development of innovative therapeutic and preventive approaches (Dasari et al., 2023; Lanz et al., 2022; Zamecnik et al., 2024).

In contrast, for AIE, risk factors and early events in pathogenesis remain less clearly defined. While extensive work has elucidated pathophysiological effects of the disease-defining anti-neuronal autoantibodies (Pruss, 2021), including both direct receptor-mediated dysfunction (Crisp et al., 2019; Moscato et al., 2014; Noviello et al., 2022) and secondary Fc-mediated immune mechanisms (Di Liberto et al., 2025; Xu et al., 2025), the initiating events leading to their formation are poorly understood.

The most common form of AIE, the anti-N-methyl-D-aspartate receptor encephalitis (NMDAR-E), has frequently been observed following herpes simplex virus encephalitis (HSE) (Armangue et al., 2018; Pruss et al., 2012), suggesting a specific link between herpes simplex virus (HSV) infection and subsequent NMDAR autoimmunity. This hypothesis was supported in a mouse model of experimental herpes simplex infection, in which four of six animals developed NMDAR autoantibodies (Linnoila et al., 2019), and in increased antibody titers in the sera of patients with NMDAR-E without clinical HSE (Salovin et al., 2018). However, the specificity of this link has been questioned by reports of anti-neuronal autoantibodies targeting non-NMDAR autoantigens in some HSE patients (Handoko et al., 2019; Mohammad et al., 2014), and of NMDAR-E cases after other viral infections (Hou et al., 2019; Luo et al., 2022; Ma et al., 2020). Thus, the co-occurrence of HSE and NMDAR-E could reflect either a pathogen-specific immunological mechanism or a non-specific immune response resulting from infection-induced immune activation and exposure of immune cells to neuronal antigens released during CNS damage (Armangue et al., 2023). Moreover, because HSE is the most common viral encephalitis and NMDAR-E is the most common form of AIE, their frequent coincidence might arise from epidemiological overlap rather than causality.

Although numerous virus-AIE associations have been described, no comprehensive analysis has systematically quantified their frequency, spectrum, or distribution across antibody-defined subtypes. Previous studies have focused on single viruses (Armangue et al., 2014, 2015; Hacohen et al., 2014; Liu et al., 2021; Luo et al., 2022; Nabizadeh et al., 2022; Tian et al., 2019), specific viral families (Linnoila et al., 2016), or selected vaccines (Samim et al., 2022), without capturing the broader landscape of virus- and vaccine-associated AIE.

To address this gap, we conducted a systematic literature review and analyzed 556 published cases of AIE associated with viral infection or antiviral vaccination, complemented by population-based viral encephalitis data. Specifically, we asked whether the co-occurrence of HSE and NMDAR-E is disproportionately frequent compared with other AIE subtypes and with epidemiological expectations from viral encephalitis cohorts. Using this dataset, we aimed to: (1) quantify and map all reported virus- and vaccine-associated AIE cases across ten antibody-defined subtypes, (2) characterize their epidemiological and clinical features, (3) compare HSV frequencies in virus-associated NMDAR-E with those in population-based viral encephalitis cohorts, and (4) examine the largest publicly available HSE-NMDAR-E cohort to explore characteristics for potential pathogen-driven mechanisms.

## 2 Materials and methods

### 2.1 Literature search strategy and selection criteria

The review was designed to quantify and compare virus- and vaccine-associated AIE cases across antibody-defined subtypes, and to evaluate whether HSV-NMDAR-E represents a disproportionate association. To this end, we conducted a systematic literature review in accordance with PRISMA (Preferred Reporting Items for Systematic Reviews and Meta-Analyses) 2020 guidelines (Page et al., 2021). The review protocol, including research questions, eligibility criteria, and data extraction variables, was defined prior to database screening and was registered as a retrospective systematic review on the Open Science Framework (https://doi.org/10.17605/OSF.IO/2AGK7).

The PubMed database was searched using predefined terms covering ten major AIE entities (last accessed on 2025-01-03). These ten forms were selected based on a comprehensive review (Dalmau and Graus, 2018), and included AIE forms defined by antibodies targeting the following autoantigens: NMDAR, Alpha-amino-3-hydroxy-5-methyl-4-isoxazolepropionic acid receptor (AMPAR), γ-Aminobutyric acid (GABA)_A_ receptor, GABA_B_ receptor, Leucine-rich glioma-inactivated 1 (LGI1), Contactin-associated protein-like 2 (CASPR2), metabotropic glutamate receptor subtype 5 (mGluR5), Dopamine D2 receptor (D2R), Dipeptidyl Peptidase-like Protein-6 (DPPX), and Neurexin-3-α. All but NMDAR were referred to as non-NMDAR entities. Each search term followed the generalized structure: (Autoantigen) AND (encephalitis OR antibody OR antibodies) AND (virus OR viral OR vaccination OR vaccine). For each AIE form, the placeholder ‘(Autoantigen)’ was replaced by the full name, abbreviation, and, when applicable, common synonyms of the respective target antigen. Searches were limited to publications after the first description year of each AIE entity, defined individually for each autoantibody. Full PubMed query strings and year ranges are listed in Supplementary Table S1.

**Fig. 1:**
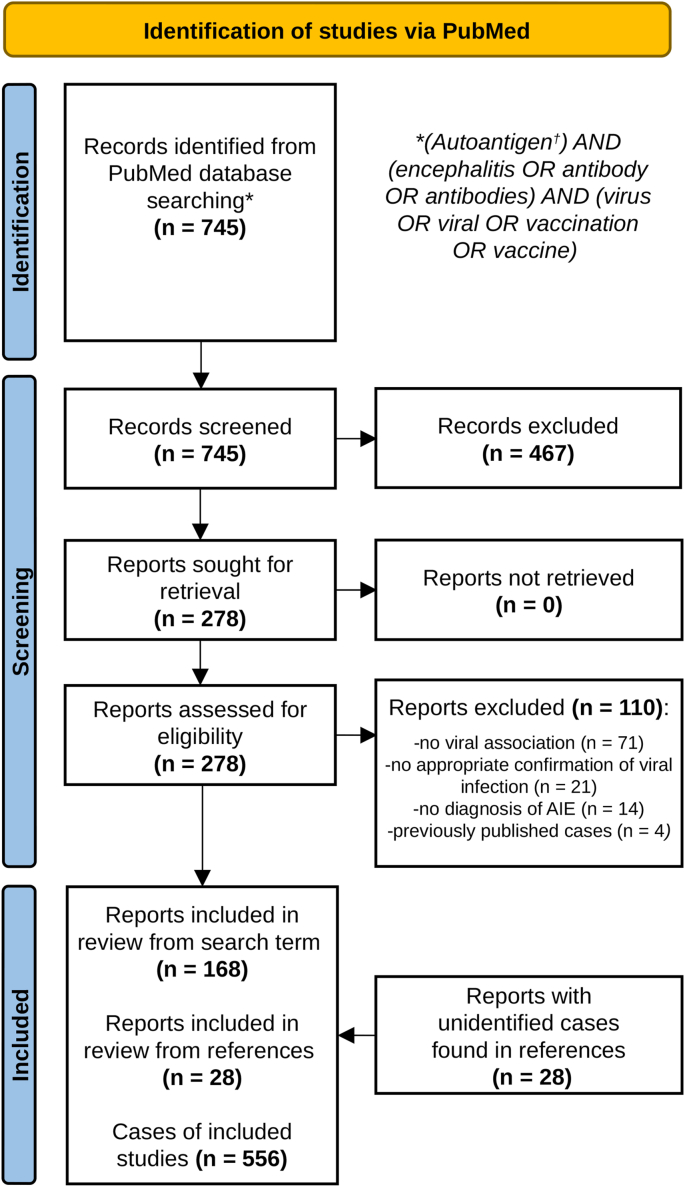
**PRISMA flow diagram for systematic review.** Flowchart of the systematic search process to identify cases of AIE associated with viral infection or antiviral vaccination, showing stages of identification, screening, and inclusion. In total, 556 individual cases from 196 reports were included in the final dataset. ∗ Search term used. † Autoantigens searched: AMPAR, CASPR2, D2R, DPPX, GABA_A_, GABA_B_, LGI1, mGluR5, Neurexin-3-α, and NMDAR (queried using both abbreviations and full names; see Supplementary Table S1 for complete strings).

The selection process, including identification, screening, eligibility, and inclusion followed PRISMA (Page et al., 2021) 2020 recommendations and is summarized in Fig. 1. In addition to the database search, we performed backward citation chasing by screening reference lists of full-text articles and related reviews to identify additional publications meeting the inclusion criteria.

The following inclusion and exclusion criteria were applied:

**Inclusion criteria**: Reports on human subjects were included if statements (1) AND (2) applied:

1. Reported clinical diagnosis or clinical phenotype consistent with AIE with detection of anti-[autoantigen∗]-autoantibodies (IgG isotype) in serum OR cerebrospinal fluid (CSF) (∗ autoantigen = NMDAR, AMPAR, CASPR2, D2R, DPPX, GABA_A_, GABA_B_, LGI1, mGluR5, or Neurexin-3α respectively).
2. A temporal association with one of the following medical conditions:
  a. Acute viral infection OR viral reactivation, verified by:
    i. Direct evidence: polymerase chain reaction (PCR) detection of viral DNA/RNA, virus isolation, or antigen detection.
    ii. Indirect evidence: Presence of virus-specific antibodies with a suitable clinical picture consistent with viral infection/reactivation.
  b. Vaccination against a viral pathogen.

A temporal association was assumed if the viral infection, reactivation, or vaccination occurred before or at the time of AIE onset, as determined by the study authors.

**Exclusion criteria:** Reports were excluded if they met any of the following conditions:

1. No viral infection or antiviral vaccination was described.
2. The viral association was insufficiently confirmed (e.g., lack of appropriate virological testing).
3. No diagnosis of AIE and/or no clinical phenotype consistent with AIE was reported.
4. Only non-IgG isotypes of the respective anti-[autoantigen]-antibodies were described.
5. The report did not include original patient data (e.g., reviews or duplicated case publications).

Articles published in languages other than English, German, French, or Spanish were translated using DeepL (*n* = 4) (https://www.deepl.com/de/translator). All PubMed records were exported to Microsoft Excel and Rayyan (https://www.rayyan.ai/) for screening and data management. Title and abstract screening were performed independently by two reviewers (S.B. and P.S.-F.) using the predefined inclusion and exclusion criteria. Each record was either excluded or marked for full-text review. Discrepancies between reviewers were resolved by discussion until consensus was reached. Full-text screening and data extraction were performed by one reviewer (S.B.) according to a predefined data extraction template. Unclear or borderline cases were discussed with the senior author (J.K.) until consensus was reached. Duplicate or overlapping case reports were identified based on overlapping clinical details, authorship, and publication data and were excluded. The full list of included and excluded reports is provided in Supplementary Table S2. No automated or AI-based tools were used at any stage of the search, screening, or extraction process.

### 2.2 Data collection

**Fig. 2:**
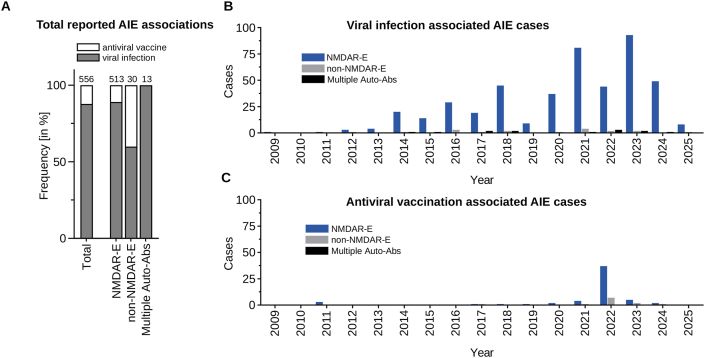
**Reported AIE cases associated with viral infection or antiviral vaccination. (A)** Stacked bar plots showing the relative frequencies of reported AIE cases in association with viral infection (*n* = 488) or antiviral vaccination (*n* = 68), stratified by NMDAR-E, non-NMDAR-E, and cases with multiple autoantibodies (Multiple Auto-Abs). *n*-values are indicated above each bar. **(B)** Stacked bar plots showing the temporal distribution of viral infection-associated AIE cases and **(C)** antiviral vaccination-associated AIE cases, shown by year of publication and stratified as in (A).

**Fig. 3:**
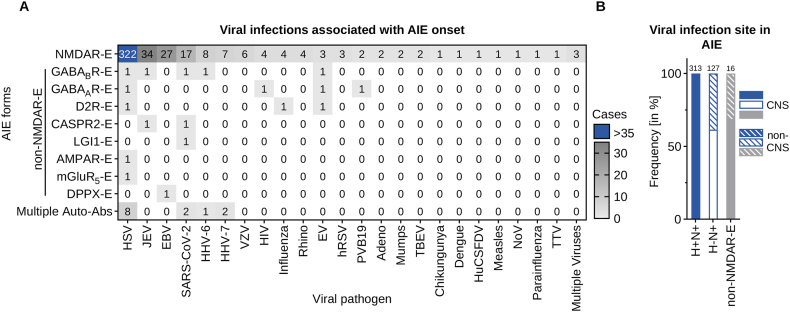
**Frequencies of viral infections associated with AIE onset. (A)** Heat map showing the absolute frequencies of detected viral species from infections reported in association with the onset of NMDAR-E (*n* = 457), non-NMDAR-E (*n* = 18), or cases with multiple autoantibodies (Multiple Auto-Abs; *n* = 13). **(B)** Stacked bar plots show the proportions of CNS and non-CNS infections among viral infection-associated AIE cases, stratified by HSV-associated NMDAR-E (H + N+), non-HSV-associated NMDAR-E (H-N+), and non-NMDAR-E AIE. CNS infections accounted for 312, 78, and 11 cases in these groups, respectively; *n*-values indicate total numbers per group and are displayed above each bar. Adeno = Adenovirus, Chikungunya = Chikungunya virus, Dengue = Dengue virus, EBV = Epstein-Barr virus, EV = Enterovirus, HIV = Human immunodeficiency virus, HHV-6 = Human herpesvirus 6, HHV-7 = Human herpesvirus 7, HuCSFDV = Human cerebrospinal fluid-associated densovirus, hRSV = Human respiratory syncytial virus, HSV = Herpes simplex virus, Influenza = Influenza virus, JEV = Japanese encephalitis virus, Measles = Measles virus, Mumps = Mumps virus, NoV = Norovirus, Parainfluenza = Human parainfluenza virus, PVB19 = Human parvovirus B19, Rhino = Rhinovirus, SARS-CoV-2 = Severe acute respiratory syndrome coronavirus 2, TBEV = Tick-borne encephalitis virus, TTV = Torque teno virus, VZV = Varicella-zoster virus.

Virus-associated AIE cohort: For all included virus- or vaccine-associated AIE cases, patient-level data were extracted, where available, based on a predefined set of variables. These included: number of cases, age, sex, pre-existing conditions, AIE form (antibody-defined entity), antibody positivity in serum and/or CSF, antibody titers in serum and/or CSF, presence of additional antibodies targeting neuronal surface proteins, associated viral infection, viral pathogen, site of infection (central or non-central), associated vaccination, time interval between viral infection or vaccination and AIE onset, symptoms at AIE onset and clinical course, and the patient's geographic origin at disease onset. The latter was recorded as indicated by the reporting authors or, if not stated, defined by the origin of the reporting institution. Where available, autoantibody positivity and titers were recorded as compartment-specific variables for CSF and serum. Given the predominance of case reports and small case series, a formal risk-of-bias assessment using standardized tools was not applicable. To ensure data reliability, we restricted inclusion to cases with confirmed IgG antibodies against one of the target antigens and a clinical phenotype compatible with AIE, together with appropriate virologic confirmation of infection or reactivation, or documented vaccination against a viral pathogen. Symptoms were grouped into 14 categories for analysis. The classification was based on reported symptom descriptions and applied consistently across all cases. The site of infection was classified as CNS infection (‘central infection’) if encephalitis was diagnosed clinically, if there was direct viral detection in the CSF, or if there was indirect viral detection in the CSF together with a compatible clinical picture. Because the serological interpretation for EBV differs from other viral infections and can reflect acute infection, reactivation, or past exposure, EBV infection status was defined separately using standard criteria (De Paschale and Clerici, 2012). Acute infection was defined by anti-VCA-IgM positivity and/or anti-VCA-IgG in the absence of anti-EBNA1-IgG, while reactivation was defined by high anti-VCA-IgG and anti-EA-IgG titers. We categorized the time intervals between viral infection or vaccination and AIE onset into three groups (0 days, 1-120 days, and >120 days) to enable a more differentiated comparison of temporal associations. For cases in which viral diagnostics and AIE onset were documented at the same time point, we further distinguished between active infection (0 days; PCR-positive at AIE onset) and recent infection (1-120 days; IgM positivity indicating recent exposure), following the conceptual approach used by Sandweiss et al. (2023). Reported intervals were used as provided in the original publications. Cases with multiple AIE-defining antibodies (*n* = 13) were shown separately as cases with multiple autoantibodies (Multiple Auto-Abs) in Fig. 2, Fig. 3A and Supplementary Fig. S2, and were excluded from further analyses as they could not be classified as either NMDAR-E or non-NMDAR-E. Similarly, cases with multiple viral infections (*n* = 3, all associated with NMDAR-E) are shown separately in Fig. 3A and Supplementary Fig. S2 and were excluded from analyses stratified by individual viral agents.

Viral encephalitis cohorts: The distribution of viral agents in the viral encephalitis populations was retrieved from 16 cohorts of infectious encephalitis reviewed by Boucher et al. (2017) and seven additional suitable studies identified through references and citations of these articles. Studies were eligible if they reported the frequencies of individual viral agents among patients with confirmed or probable viral encephalitis. For each study, infectious agents were classified as HSV or non-HSV and, if reported, further subclassified as HSV-1 or HSV-2. Information on recruitment criteria was extracted for each cohort, including geographic origin (continent) and age category (defined as children ≤17 years, adults >17 years, or both).

### 2.3 Statistical analysis

For statistical analysis and illustrations GraphPad Prism in version 10.6.0 was used. For comparison of HSV frequency within the distribution of viral agents, all viral encephalitis studies were first combined and compared with all reviewed cases of NMDAR-E in association with viral CNS infection. For subgroup analyses, each viral encephalitis study was then compared separately with the corresponding subgroup of NMDAR-E cases associated with viral CNS infection, stratified by the same geographic category at the continent level (Europe, Asia, North America, South America, Australia, or Africa) and the same age category (children ≤17 years, adults >17 years, or both), as detailed in Supplementary Table S3. Confidence intervals were computed using the Wilson/Brown method (Brown et al., 2001). Groupwise frequencies were compared using two-sided chi^2^ tests, and antibody titers with two-tailed Mann-Whitney tests. For multiple comparisons, *P*-values were adjusted using the Benjamini-Hochberg false discovery rate (FDR) correction. Formal assessment of publication bias (e.g., funnel plots or small-study effects) was not feasible due to the case-based nature of the dataset without comparable effect estimates.

## 3 Results and discussion

### 3.1 HSV-associated NMDAR-E outnumbers the total of all other virus-AIE combinations

To quantify AIE cases with disease onset in association with viral infections or antiviral vaccinations, we performed a systematic literature review across 10 common forms of AIE (Fig. 1). This yielded 556 viral infection- or vaccine-associated AIE cases, including 513 NMDAR-E, 30 non-NMDAR-E, and 13 cases with multiple anti-neuronal autoantibodies (Multiple Auto-Abs) (Fig. 2A). Most cases were linked to viral infections (87.8%, *n* = 488), whereas fewer followed antiviral vaccinations (12.2%, *n* = 68) (Fig. 2A). Reported infection-associated cases increased gradually over time (Fig. 2B). Vaccination-associated cases, although rare, showed a distinct reporting peak in 2022 (Fig. 2C), potentially reflecting heightened clinical awareness and surveillance during the SARS-CoV-2 pandemic and global vaccination campaigns (Hosseini and Askari, 2023; Nazar et al., 2025; van der Boor et al., 2023).

Vaccination-associated AIE onset was reported following eight different vaccines or vaccine combinations (Supplementary Fig. S1A), with a median interval of 14 days (IQR 6-27) between vaccination and first symptoms, and with most cases occurring within 30 days (Supplementary Fig. S1B). No single vaccine predominated among NMDAR-E cases. In contrast, post-vaccination LGI1-E cases (*n* = 8) were exclusively reported after SARS-CoV-2 vaccination. Differences in age distribution may have contributed to this pattern, as LGI1-E typically affects older adults, who may have different vaccination exposure patterns than younger adults and children, in whom NMDAR-E is more frequent. Given the very small number of post-vaccination LGI1-E cases, this pattern might reflect reporting bias or temporal clustering rather than a specific causal association, though continued surveillance remains warranted. Taken together, vaccine-associated AIE cases were extremely rare in the published literature, and the available case-based evidence is insufficient to infer causality beyond temporal association. Consequently, our data do not support causal inference and instead provide a descriptive overview of published temporal associations between antiviral vaccinations and AIE onset. This cautious interpretation is consistent with a large population-based study that did not identify an overall association between SARS-CoV-2 vaccination and 28-day composite neurological outcomes of encephalitis, meningitis, or myelitis, whereas this outcome was associated with SARS-CoV-2 infection itself (Patone et al., 2021).

Regarding viral infections or reactivations, we identified 488 cases of 23 distinct viruses associated with AIE onset (Fig. 3A, references listed in Supplementary Table S2). In the NMDAR-E group (*n* = 457), associations were most frequently reported with HSV (70.5%, *n* = 322), followed by Japanese encephalitis virus (JEV) (7.4%, *n* = 34) and EBV (5.9%, *n* = 27). All other viral associations individually accounted for less than 4% of infection-associated NMDAR-E cases. In the non-NMDAR-E group (*n* = 18), HSV infections were likewise the most frequent (27.8%, *n* = 5), followed by enteroviruses (*n* = 3), SARS-CoV-2 (*n* = 3) and JEV (*n* = 2), with all other virus-AIE combinations reported only once (Fig. 3A).

To assess the temporal relationship between infection and AIE onset, we stratified all virus-AIE associations by the reported interval between infection and AIE onset (Supplementary Fig. S2). Within the group of concurrent infection and AIE diagnosis (interval 0 days), cases (*n* = 39, 10.7%) were most commonly associated with EBV (*n* = 9), HHV-6 (*n* = 6), and HSV (*n* = 6) (Supplementary Fig. S2A). The group of infections preceding AIE onset (interval 1-120 days) contained the majority of cases (*n* = 307, 84.3%), and the distribution of virus-AIE combinations was broadly consistent with the overall cohort. HSV and NMDAR-E remained the most frequent association, followed by JEV preceding NMDAR-E (Supplementary Fig. S2B). Interestingly, EBV no longer ranked among the three leading associations, since EBV was predominantly reported in cases with concurrent viral detection and AIE diagnosis. Late-onset AIE (>120 days after viral infection) was rarely reported (*n* = 18, 4.9%) (Supplementary Fig. S2C).

Notably, all but one HSV infection case linked to NMDAR-E (H+N+ cases with available data on site of infection, *n* = 313) were described as infections of the CNS (encephalitis or meningoencephalitis), despite HSV being a common cause of mucocutaneous infections in humans (Whitley and Roizman, 2001) (Fig. 3B). In contrast, NMDAR-E associated with non-HSV viruses (H-N+, *n* = 127) and the smaller non-NMDAR-E cohort (*n* = 16) included a substantial proportion of non-CNS infections (*n* = 49, 38.6%; and *n* = 5, 31.3%, respectively), most frequently from the respiratory tract (Fig. 3B).

### 3.2 Disproportionate enrichment of HSE in NMDAR-E

To determine whether the frequent co-occurrence of HSE and NMDAR-E exceeds background epidemiology, we performed two complementary disproportionality analyses: (i) a within-AIE comparison of HSV frequency between NMDAR-E and non-NMDAR-E subtypes, and (ii) a population-level comparison between CNS viral-infection-associated NMDAR-E and 23 published viral encephalitis cohorts across five continents.

First, we compared the frequency of HSV among different AIE subtypes within our cohort. Although several non-HSV viruses were reported in association with NMDAR-E, and HSV was also the most common viral trigger among non-NMDAR forms of AIE, the combination of HSV infection and NMDAR-E occurred markedly more often. Across all viral infections, 70.6% of NMDAR-E cases versus 27.8% of non-NMDAR-E cases were linked to HSV (*P* = 0.0001), and the difference remained significant when restricting the analysis to CNS infections (80.0% vs. 36.4%, *P* = 0.0005) (Table 1). These findings indicate that the HSE-NMDAR-E association exceeds what would be expected from the simple convergence of the most common viral encephalitis and the most frequent form of AIE.

**Table 1:** Relative frequencies of HSV infections in NMDAR-E and non-NMDAR-E.

AIE group	Frequency of HSV among all viral infection-associated AIE cases	Frequency of HSV among all viral CNS infection-associated AIE cases
NMDAR-E	70.6% (*n* = 456)	80.0% (*n* = 390)
non-NMDAR-E	27.8% (*n* = 18)	36.4% (*n* = 11)
*P*-value from chi^2^	0.0001	0.0005

Frequencies of HSV infections among viral infection-associated NMDAR-E and non-NMDAR-E cases are shown with corresponding *P*-values from chi^2^ tests. Comparisons were performed separately for AIE cases associated with any viral infection (left column) and for those with confirmed viral infection of the CNS (right column). The displayed *n*-values indicate the total number of AIE cases per category.

**Fig. 4:**
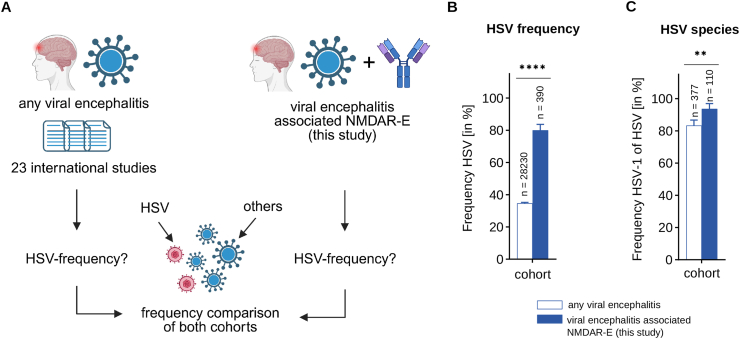
**Relative frequencies of HSV infections in viral encephalitis cohorts and viral CNS infection-associated NMDAR-E. (A)** Schematic overview of the comparative analysis between published population-based viral encephalitis cohorts and literature-derived central viral infection-associated NMDAR-E cases (here simplified as ‘viral encephalitis-associated NMDAR-E’). Created in BioRender. Kreye, J. (2026) https://BioRender.com/tcbzp8y. **(B)** Stacked bar plots show the frequencies of HSV infections among viral encephalitis patients (combined data from 23 studies across five continents) and among NMDAR-E cases associated with viral CNS infection. *n*-values indicate total numbers per group and are displayed above each bar. Bars indicate frequency +95% confidence intervals. For comparison, a chi^2^ test was used, ∗∗∗∗*P* < 0.0001. **(C)** Stacked bar plots show the frequency of HSV-1 species among patients analyzed in (B). Note that data on HSV species were available for <4% of viral encephalitis cases in population-based studies. *n*-values indicate total numbers per group and are displayed above each bar. Bars indicate frequencies +95% confidence intervals. For comparison, a chi^2^ test was used, ∗∗*P* < 0.01.

Second, to further determine whether this apparent overrepresentation persists when compared with population-based viral encephalitis cohorts, we analyzed HSV frequencies in our viral-CNS-infection-associated NMDAR-E cohort against those reported in 23 population-based studies of viral encephalitis from five continents (Fig. 4A; Supplementary Table S3). Among the 23 studies, HSE was identified as the leading or co-leading cause of viral encephalitis in 15 (65.2%) cohorts, with a combined frequency of 34.7% (*n* = 9789/28,230 cases). In contrast, HSV accounted for 80.0% (*n* = 312/390) among NMDAR-E cases that were associated with viral CNS infections in our dataset (Fig. 4B), a significantly higher proportion (*P* < 0.0001). Interestingly, when each viral encephalitis study cohort was compared with an age- and geographically matched subgroup of our viral-CNS-infection-associated NMDAR-E cohort, lower HSV frequencies were observed in the population-based viral encephalitis cohorts in all comparisons, with all but one reaching statistical significance (Supplementary Table S3).

Analysis of HSV species further supported this disproportionate relationship. HSV-1 predominated in both datasets but was significantly more frequent in the HSE-associated NMDAR-E cohort (93.6%, *n* = 103/110) than among HSE cases reported in the population-based viral encephalitis cohorts (83.3%, *n* = 314/377) (Fig. 4C). Collectively, these analyses demonstrate that HSV (particularly HSV-1) is significantly overrepresented among viral-CNS-associated NMDAR-E compared with both other AIE subtypes and general viral encephalitis populations, supporting a pathogen-specific link between HSE and NMDAR-E rather than a coincidental overlap.

### 3.3 HSE-associated NMDAR-E reflects features of post-infectious autoimmunity

**Fig. 5:**
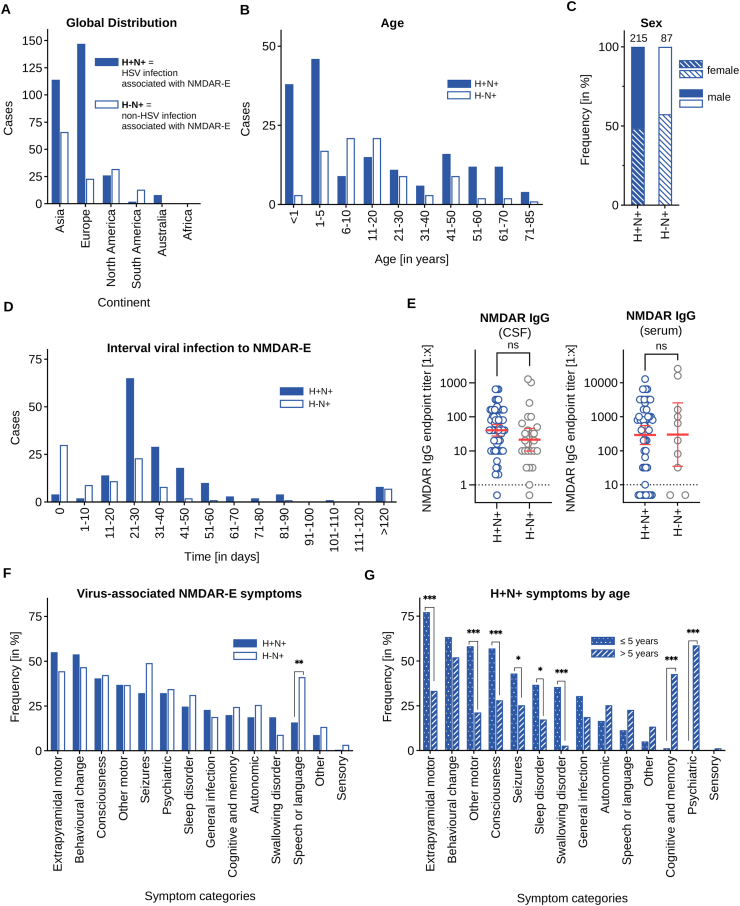
**Characteristics of NMDAR-E cases associated with HSV (H + N+) or non-HSV (H-N+) infection. (A)** Stacked bar plots show the global distribution by continent for the H+N+ group (*n* = 297) and the H-N+ group (*n* = 134). South America includes cases from Central America; Australia includes cases from Oceania. **(B)** Stacked bar plots show the age distribution for H + N+ (*n* = 188) and H-N+ cases (*n* = 88). **(C)** Stacked bar plots show the relative sex distribution for H+N+ and H-N+ cases. *n*-values are indicated above each bar. **(D)** Stacked bar plots show the time interval between viral infection and onset of NMDAR-E for H + N+ (*n* = 160) and H-N+ cases (*n* = 92). **(E)** Scatter dot plots show endpoint titer dilutions of NMDAR IgG antibodies in CSF (left) and serum (right) for the H+N+ group (*n* = 70) and the H-N+ group (*n* = 25). Bars indicate geometric mean ± 95% confidence interval. For comparison, two-tailed Mann-Whitney tests were used; ns = not significant. The dotted lines indicate the detection threshold. Negative values were set to 0.5 (CSF) or 5 (serum). **(F)** Bar plots show relative symptom frequencies within H + N+ (*n* = 171) and H-N+ (*n* = 90) cases. For comparison, chi^2^ tests with Benjamini-Hochberg correction for multiple comparisons were used and adjusted *P*-values are shown with significance levels indicated as follows: ∗*P* < 0.05, ∗∗*P* < 0.01, ∗∗∗*P* < 0.001. Results without asterisks are not significant. **(G)** Bar plots show relative symptom frequencies within the H+N+ group, comparing patients aged ≤5 years (*n* = 79) and >5 years (*n* = 75). ‘Other motor symptoms’ include all non-extrapyramidal motor deficits (e.g., paresis). Statistical comparison performed as in (F).

To further characterize the patient subgroup of NMDAR-E that is associated with HSV infection (H + N+, *n* = 322), we compared these cases with NMDAR-E associated with non-HSV infection (H-N+, *n* = 134). Most H+N+ cases were reported from Europe (Fig. 5A) (*n* = 147/297, 49.5%), primarily driven by a large Spanish cohort (Armangue et al., 2014, 2015, 2018, 2023). In contrast, most H-N+ cases originated from Asia (*n* = 66/134, 49.3%) (Fig. 5A), largely reflecting the high prevalence of JEV cases in that region (Campbell et al., 2011). Viral infection-associated non-NMDAR-E cases were reported globally, with Europe and Asia predominating (Supplementary Fig. S3A).

Differences in geographic distribution may partly reflect genetic factors influencing susceptibility. For instance, Armangué et al. (Armangue et al., 2023) reported a lower frequency of the human leukocyte antigen (HLA) A∗02 allele in post-HSE AIE (with predominantly NMDAR-E cases). However, since this allele is not less common in Spanish or European populations (Arrieta-Bolanos et al., 2023), the geographic clustering of H+N+ cases is unlikely to be explained by HLA distribution alone. In addition, geographic clustering may also reflect non-biological confounders, including regional differences in diagnostic practices, clinical awareness of post-infectious AIE, and publication patterns.

The age range of viral infection-associated AIE spanned from 2 months to 82 years. H+N+ patients were markedly younger (median 6 years, IQR 1-42.5) with 70.5% (*n* = 208) aged ≤18 years, indicating a pronounced early-childhood predominance. In contrast, NMDAR-E cases with associated non-HSV viral infections (H-N+ group) were older (median 11 years, IQR 6-22) and peaked among older children and adolescents (Fig. 5B) with 66.1% (*n* = 84) aged ≤18 years. This distribution differs from the overall NMDAR-E population, which shows a higher median age (20 years) and fewer pediatric cases (46.3%, *n* = 707/1526) (Nosadini et al., 2021). Notably, HSE itself shows a characteristic bimodal distribution, affecting both young children and older adults (>50 years) (Whitley and Gnann, 2002). The predominance of young patients in the H+N+ cohort, despite high HSV seroprevalence in older age groups (Bradley et al., 2014; Yousuf et al., 2020), may suggest that pre-existing HSV immunity confers protection against post-infectious autoimmunity, whereas primary infections more readily trigger secondary NMDAR-E. Possible protective mechanisms of pre-existing HSV immunity may include attenuation of prolonged interferon responses that are associated with secondary AIE after HSE (Armangue et al., 2023) and immunological imprinting that might limit the generation of unfavorable (auto-)antibodies upon re-exposure to HSV, as observed for other viral infections (Changrob et al., 2025; Gostic et al., 2016; Park et al., 2022). Future studies should investigate whether the presence of pre-existing HSV antibodies at onset of HSE correlates with a lower risk of developing secondary NMDAR-E. Viral infection-associated non-NMDAR-E cases showed a broader age range without a clear early-childhood peak (Supplementary Fig. S3B). Regarding the sex distribution, H+N+ cases were evenly balanced (48.4% female, 51.6% male), resembling general HSE populations (George et al., 2014), whereas H-N+ cases showed a mild female predominance (57.5% female, 42.5% male) (Fig. 5C). Viral infection-associated non-NMDAR-E cases showed a modest male predominance (41.2% female, 58.8% male). The observed pattern in the NMDAR-E cases of our data set (H+N+ and H-N+) differs from the overall NMDAR-E population, where a pronounced female predominance (73.3%, *n* = 707/1526) is largely driven by the association with ovarian teratoma or other ovarian tumors (21.3%, *n* = 324/1524) (Nosadini et al., 2021), which were almost absent in our infection-associated cohort (1.8%, *n* = 8/457).

We next analyzed the temporal relationship between viral infection and AIE onset. Viral infections occurred within 60 days of AIE onset in 88.8% of H + N+, 91.3% of H-N+ and 75.0% of non-NMDAR-E cases (Fig. 5D, Supplementary Fig. S3C). For the H+N+ cases, the median interval between HSE and NMDAR-E onset was 30 days (IQR 24-42), forming a characteristic bell-shaped distribution (Fig. 5D). Consistent with previous reports (Armangue et al., 2018), the latency from HSE to NMDAR-E onset was shorter in younger children (median 26 days for ≤5 years) than in older patients (median 40 days for >5 years, *P < *0.0001), suggesting a more rapid post-infectious immune response in early childhood. By contrast, intervals between viral infection and AIE onset were shorter in the H-N+ (median of 16 days, IQR 0-29) and non-NMDAR-E cases (median of 26 days, IQR 8.75-38.5). Concurrent detection of viral infection and AIE onset occurred in 32.6% of H-N+ and 12.5% of non-NMDAR-E cases, but only in 2.5% of H+N+ cases (Fig. 5D, Supplementary Fig. S3C). The most common concurrently detected virus of the H-N+ group was EBV (30.0%, n = 9/30), frequently discussed as viral reactivation rather than primary infection (Garre et al., 2019; Konen et al., 2019). Both virus reactivations and primary viral infections that were concurrently detected with AIE onset could represent a temporal coincidence rather than a causal relation. In the investigated forms of AIE, anti-neuronal IgG autoantibodies represent disease hallmarks and directly mediate pathogenic effects (Kornau et al., 2020; Kreye et al., 2016, 2021; van Hoof et al., 2024). Considering that IgG antibody production typically requires one to two weeks for detectable formation (Chang et al., 2021; Hsueh et al., 2004) and even longer for clinical manifestation (Pisetsky, 2023), simultaneous detection of infection and AIE onset likely reflects a temporal coincidence or non-specific immune activation that unmasked a subclinical autoimmunity. Conversely, the median 30-day interval between HSE and NMDAR-E onset is consistent with the expected timeframe for *de novo*, infection-triggered secondary autoimmunity (Wang et al., 2021).

Comparison of NMDAR IgG antibody titers in CSF and serum revealed no significant difference between the groups (Fig. 5E). Clinically, both H + N+ (*n* = 171) and H-N+ (*n* = 90) patients displayed the characteristic neuropsychiatric and neurological spectrum of NMDAR-E, including psychiatric symptoms, seizures, movement disorders, and autonomic dysfunction (Dalmau et al., 2008). Among individual symptoms, only speech or language disorders differed significantly, occurring more frequently in H-N+ than in H+N+ patients (41.1% vs. 15.8%, adjusted *P* < 0.01) (Fig. 5F). Other trends included higher seizure frequency in H-N+ and more extrapyramidal symptoms (most commonly presented as choreoathetosis) and swallowing disturbances in H + N+. Within the H+N+ group, symptom profiles varied markedly with age. Patients aged ≤5 years (*n* = 79) more frequently exhibited swallowing disturbances, consciousness disturbances, other motor symptoms, extrapyramidal motor symptoms, seizures and sleep disturbances. In contrast, older patients (>5 years, *n* = 75) presented predominantly with psychiatric symptoms and cognitive and memory impairment (Fig. 5G). This age-dependent clinical shift mirrors findings from prospective studies of post-HSE NMDAR-E (Armangue et al., 2018), which described that post-HSE AIE presents commonly with choreoathetosis, impaired consciousness, dysphagia and frequent refractory seizures in infants and young children, whereas older patients develop predominantly behavioral and psychiatric manifestations. Our pooled analysis corroborates this pattern with statistical significance and extends previous observations by identifying additional age-related differences in cognitive, motor, and sleep-related features. Psychiatric and cognitive symptoms may nonetheless be underestimated due to limited evaluability in very young children.

### 3.4 Characteristics of NMDAR-E associated with JEV, EBV, and SARS-CoV-2 infections

**Fig. 6:**
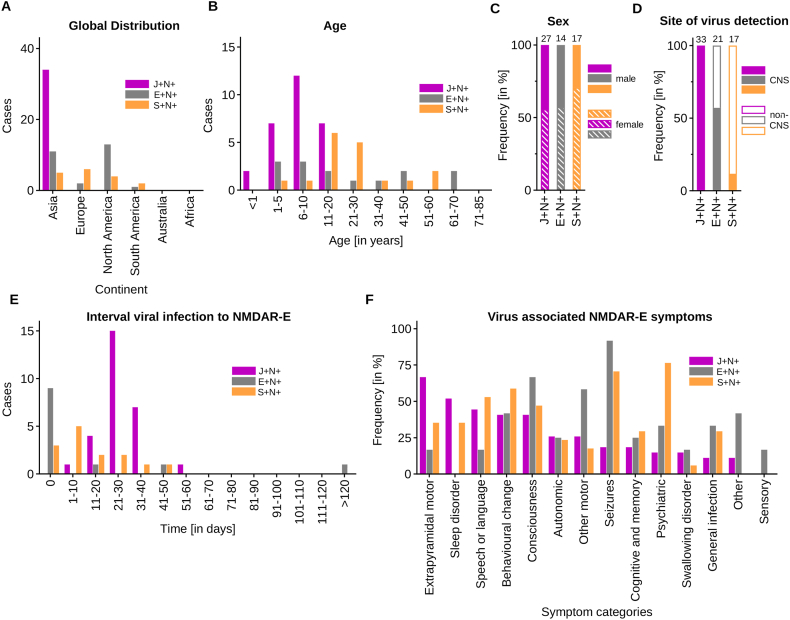
**Characteristics of NMDAR-E cases associated with JEV (J + N+), EBV (E + N+) and SARS-CoV-2 (S + N+) infection. (A)** Stacked bar plots show the global distribution by continent for J + N+ (*n* = 34), E + N+ (*n* = 27), and S + N+ (*n* = 17) cases. South America includes cases from Central America. **(B)** Stacked bar plots show the age distribution for J + N+ (*n* = 28), E + N+ (*n* = 14), and S + N+ (*n* = 17) cases. **(C)** Stacked bar plots show the relative sex distribution for J + N+, E + N+, and S+N+ cases. *n*-values are indicated above each bar. **(D)** Stacked bar plots show the site of viral detection (CNS vs. non-CNS) for J + N+, E + N+, and S+N+ cases. *n*-values are indicated above each bar. **(E)** Stacked bar plots show the time interval between viral infection and onset of NMDAR-E for J + N+ (*n* = 28), E + N+ (*n* = 12), and S + N+ (*n* = 14) cases. **(F)** Bar plots show relative symptom frequencies of J + N+ (*n* = 27), E + N+ (*n* = 12), and S + N+ (*n* = 17) cases. EBV = Epstein-Barr virus, JEV = Japanese encephalitis virus, SARS-CoV-2 = Severe acute respiratory syndrome coronavirus 2.

To assess whether other viral infections reproduce the features of secondary, infection-triggered NMDAR-E observed after HSE, we next analyzed NMDAR-E cases linked to JEV (J + N+), EBV (E + N+), and SARS-CoV-2 (S + N+), representing the three most frequent non-HSV viral associations in our dataset (Fig. 6). These subgroups showed distinct geographic distributions, with J+N+ cases reported exclusively from Asia, E+N+ cases predominantly from Asia and North America, and S+N+ cases from multiple continents (Fig. 6A). Age profiles differed across groups: J+N+ cases occurred exclusively in children (median 7 years), whereas E+N+ and S+N+ cases affected older individuals (median 15 and 21 years, respectively) (Fig. 6B). Sex distribution was balanced across all groups, with a slight female predominance in the S+N+ cases (Fig. 6C).

The anatomical distribution of viral detection sites also differed among pathogens. JEV was detected exclusively in the CNS, EBV in both central and peripheral compartments, and SARS-CoV-2 predominantly outside the CNS (Fig. 6D). The median interval between viral infection and NMDAR-E onset was 28 days for JEV, 0 days for EBV, and 10 days for SARS-CoV-2 (Fig. 6E). Thus, J+N+ cases resembled H+N+ cases in several aspects: a median latency of approximately one month between infection and NMDAR-E onset, exclusive CNS involvement and a pediatric predominance – all consistent with features of secondary, infection-triggered autoimmunity. Although a recent study identified a sequence homology between the JEV envelope protein and NMDAR epitopes, immunization with this peptide failed to elicit NMDAR antibodies in mice (Luo et al., 2024), arguing against molecular mimicry as the underlying mechanism.

In E+N+ cases, infection and NMDAR-E onset were often concurrent, making it difficult to determine causality. Reported EBV associations were mainly serological rather than PCR-based (20/27), in contrast to HSE-associated NMDAR-E, where HSV infection is typically supported by PCR confirmation in the CSF. EBV detection may thus reflect either viral reactivation secondary to immune activation during NMDAR autoimmunity, or a coincidental infection temporally overlapping with AIE onset. S+N+ cases generally occurred after short intervals following peripheral infection. Given the high global prevalence of SARS-CoV-2 and widespread diagnostic testing during the COVID-19 pandemic, this association may largely reflect coincidental overlap (Vasilevska et al., 2024). Less likely, it may reflect nonspecific immune activation amplifying a pre-existing autoimmunity rather than a specific causal trigger. Reported clinical features were heterogeneous across viral groups. J+N+ cases showed a tendency towards extrapyramidal motor symptoms, E+N+ cases towards seizures and disturbances of consciousness, and S+N+ cases towards psychiatric symptoms accompanied by seizures (Fig. 6F).

Collectively, these data suggest that CNS-restricted infections, notably JEV and HSV, consistently exhibit epidemiological hallmarks suggestive of secondary, infection-triggered NMDAR autoimmunity: a pediatric predominance, exclusive CNS involvement, and a latency of approximately one month. In contrast, EBV- and SARS-CoV-2-associated cases often coincide temporally with AIE onset and are likely to reflect viral reactivation or non-specific immune amplification rather than causal triggers.

### 3.5 Mechanisms linking HSE to secondary NMDAR-E remain undefined

**Fig. 7:**
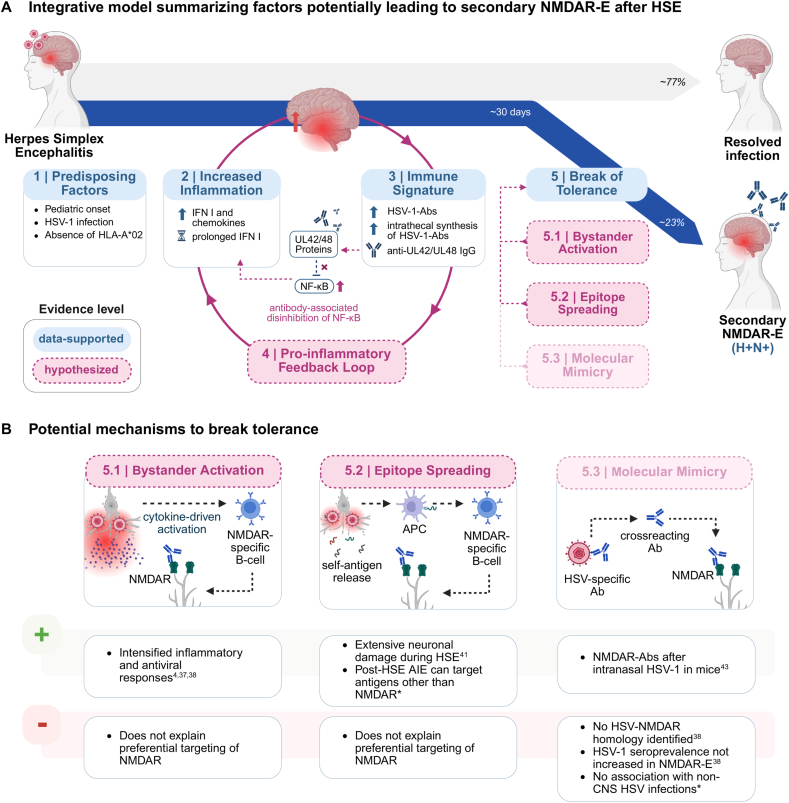
**Proposed pathways linking HSE to secondary NMDAR encephalitis.** Created in BioRender. Kreye, J. (2026) https://BioRender.com/v6120r9**(A)** An integrative model summarizing data-supported observations (blue) and hypothesized steps requiring further validation (pink) in the development of secondary NMDAR-E after HSE. Predisposing host factors (1), exaggerated CNS inflammation (2), and amplified HSV-specific humoral responses (3) converge to lower tolerance thresholds and enable anti-NMDAR IgG production (5). Distinct UL42/UL48-directed “signature antibodies” may relieve UL42/UL48-mediated inhibition of NF-κB/type I interferon signaling, sustaining inflammatory feedback (4). **(B)** Candidate mechanisms for tolerance break after HSE include bystander activation, epitope spreading, and molecular mimicry. Plus/minus symbols highlight key observations supporting or limiting each mechanism. Numerical labels retained in the figure correspond to the following references: 4 = Armangue et al., 2023; 37 = Kothur et al., 2017; 38 = Kreye et al., 2025; 41 = Kumar et al., 2024; 43 = Linnoila et al., 2019;∗ this study. Ab = antibody, APC = antigen-presenting cell, HLA = human leukocyte antigen, IFN I = type I interferon. (For interpretation of the references to colour in this figure legend, the reader is referred to the Web version of this article.)

Despite these characteristic clinical and epidemiological features of post-HSE NMDAR-E, the immunopathogenic pathways linking HSE to secondary autoimmunity remain incompletely understood. Fig. 7 presents a hypothesis-generating working model that distinguishes evidence-supported observations from speculative mechanistic steps requiring further experimental validation. The model proposes that, in a subset of HSE patients, predisposing host factors, increased CNS inflammation, and amplified HSV-specific humoral responses converge to lower the threshold for loss of immune tolerance and anti-NMDAR antibody production as the biological hallmark of NMDAR-E.

Predisposing host factors appear to modify susceptibility to post-HSE autoimmunity. While multiple toll-like receptor 3-interferon pathway mutations confer risk to HSE itself (Andersen et al., 2015; Bourdon et al., 2020; Mork et al., 2015), these variants are not enriched among patients who develop AIE secondary to HSE (Armangue et al., 2023), suggesting that primary genetic defects in antiviral sensing do not drive progression toward autoimmunity. Instead, reported predisposing features in post-HSE NMDAR-E include pediatric onset, HSV-1 infection, and reduced HLA-A∗02 frequency (Fig. 7A, step1) (Armangue et al., 2023). These factors may influence the magnitude and quality of antiviral immune responses within the CNS.

Consistent with an exaggerated inflammation, post-HSE AIE patients show intensified and prolonged interferon-pathway activation (Armangue et al., 2023) and elevated Th1- and B-cell-associated chemokines (e.g. CXCL9/10/13, CCL19, APRIL) (Fig. 7A, step 2) (Kothur et al., 2017)**.** Likewise, higher HSV-1 antibody titers and greater intrathecal antibody synthesis distinguish HSE patients who develop NMDAR-E from those who recover without autoimmunity (Kreye et al., 2025), consistent with more sustained antiviral immune activation within the CNS (Fig. 7A, step 3). Collectively, these observations suggest a prolonged pro-inflammatory milieu capable of lowering tolerance thresholds, similar to mechanisms described in systemic lupus erythematosus (Baechler et al., 2003) and genetic type I interferonopathies (Crow and Stetson, 2022). A potential self-reinforcing feedback loop may amplify this response (Fig. 7A, step 4). Specific antibodies targeting HSV-1 regulatory proteins UL42 and UL48 have recently been identified as a serological signature for post-HSE NMDAR-E (Kreye et al., 2025). Because UL42 and UL48 normally inhibit NF-κB and interferon pathways (Chapon et al., 2019; Xing et al., 2013; Zhang et al., 2013), such antibodies could relieve this viral suppression, thereby sustaining cytokine release and inflammatory signaling (Fig. 7A,step 4). Such reinforcement could further promote a CNS environment conducive to tolerance break, culminating in NMDAR-directed autoimmunity (Fig. 7A,step 5).

Several downstream mechanisms for tolerance break following (viral) infections are known from other post-infectious autoimmune disorders and have been proposed also in the context of post-HSE NMDAR-E (Pruss, 2021). These include bystander activation, epitope spreading and molecular mimicry (Fig. 7A, step 5 and 7B). Inflammatory cytokines released during HSE may transiently disrupt the blood-brain barrier and activate low-frequency autoreactive lymphocytes (bystander activation) (Fig. 7B, mechanism 5.1). Virus-induced neuronal destruction during HSE (Kumar et al., 2024) may expose previously sequestered CNS antigens, enabling antigen drainage to deep cervical lymph nodes where germinal center reactions support affinity maturation and class switching (epitope spreading) (Fig. 7B, mechanism 5.2) (Blackburn and Wang, 2020; Cleaver et al., 2024; Sun et al., 2020). Molecular mimicry describes structural similarities between viral and self-antigens that elicit cross-reactive (auto)antibodies (Fig. 7B, mechanism 5.3), a known mechanism from other autoimmune diseases such as rheumatic fever (Cunningham et al., 1989), neurological Guillain-Barré syndrome (Yuki et al., 2004) and more recently, in MS (Lanz et al., 2022). For the link between HSE and NMDAR-E, molecular mimicry remains unsubstantiated. While intranasal HSV-1 infection induced NMDAR antibodies in a subset of mice (Linnoila et al., 2019), large NMDAR-E cohorts without HSE showed HSV-1 seroprevalence, titers, and intrathecal synthesis comparable to controls (Kreye et al., 2025), and no HSV/NMDAR epitope similarity has been demonstrated (Fig. 7B,mechanism 5.3). If molecular mimicry were the dominant mechanism, one would expect to find links between HSV immunity and NMDAR autoimmunity also following systemic or mucocutaneous HSV infection independent of CNS involvement; instead, secondary NMDAR-E has been reported almost exclusively after HSE. The greater plausibility of epitope spreading and bystander activation relative to molecular mimicry is consistent with the observation that HSE can trigger AIE with antibodies against other neuronal antigens (Fig. 3A), as both mechanisms are antigen-independent. Yet these pathways alone do not readily explain the disproportionate enrichment of NMDAR-E among post-HSE AIE, underscoring the need to define additional determinants of antigen specificity in this setting. Taken together, current data support a model in which CNS-localized HSV infection, prolonged interferon-mediated inflammation, and intensified HSV-specific immunity generate a permissive environment for loss of tolerance and anti-NMDAR antibody production (Fig. 7A). This framework aligns with the key epidemiological features of post-HSE NMDAR-E: exclusive CNS infection, pediatric predominance and characteristic latency of approximately one month.

### 3.6 Limitations

This study has several limitations. First, because our dataset was primarily derived from published case reports and case series, with only a few systematic or prospective studies available, the findings are subject to publication bias. Rare or unusual virus-AIE associations may have been preferentially reported, whereas more common or expected combinations could be underrepresented. Previously reported associations may also have influenced subsequent diagnostic testing and reporting practices, thereby reinforcing already established virus-AIE links in the published literature, such as post-HSE NMDAR-E. However, the absence of denominator populations, systematic testing rates, and unpublished negative cases precluded formal assessment of temporal reporting trends. Second, the distribution of viral associations may have been influenced by diagnostic bias, as pathogens more frequently included in diagnostic panels are more likely to be detected and reported, potentially overestimating their apparent contribution to AIE. In addition, mild or asymptomatic viral infections or reactivations may remain clinically unrecognized and therefore untested, leading to underrepresentation of such infections. Third, the comparison of HSV frequencies (Fig. 4, Supplementary Table S3) relied on two distinct data sources: literature-derived virus-associated NMDAR-E cases with inherent selection and reporting bias, and population-based epidemiological studies of viral encephalitis. While these methodological differences complicate direct comparisons of frequencies, this combined approach nevertheless enabled assessment of whether HSE is disproportionately associated with NMDAR-E. Fourth, the temporal association between SARS-CoV-2 vaccination and AIE may have been influenced by the COVID-19 pandemic, which not only expanded global vaccination coverage but also heightened clinical vigilance and reporting of potential adverse events. Finally, our systematic review focused on ten established antibody-defined AIE subtypes. Post-viral autoantibodies targeting novel or incompletely characterized brain antigens beyond these subtypes were therefore not systematically captured. Although their pathogenic and diagnostic significance may be less certain, such cases may represent an important component of post-infectious brain autoimmunity and warrant dedicated future studies.

## 4 Conclusion

In this study, we provide the most comprehensive overview to date of viral infection- and antiviral vaccination-associated AIE, encompassing 556 published cases across ten antibody-defined subtypes. This includes the largest publicly available cohort of HSE-associated NMDAR-E. Across this global dataset, four converging observations support a pathogen-specific association between HSV and secondary NMDAR-E, summarized in four major observations:

(i) HSV-NMDAR-E cases outnumber the total of all other reported virus-AIE combinations (Fig. 3A).
(ii) HSV infections are significantly overrepresented among NMDAR-E compared with non-NMDAR-E cases (Table 1).
(iii) HSE occurs more frequently in virus-associated NMDAR-E than in population-based viral encephalitis cohorts from five continents (Fig. 4B, Supplementary Table S3).
(iv) The epidemiological characteristics with pediatric predominance, CNS restriction, and median 30-day latency of the H+N+ cohort are consistent with a secondary, infection-triggered autoimmune mechanism (Fig. 3B; Fig. 5B and D).

Together, these findings delineate HSV-associated NMDAR-E as the predominant and disproportionately frequent virus-AIE association worldwide, pointing to a pathogen-specific principle rather than coincidental overlap. Future research should aim to elucidate the immunological and genetic mechanisms underlying this link, identify and advance biomarkers predictive of secondary NMDAR-E, and translate these insights into early risk stratification and preventive treatment after HSE. Early identification of patients at risk may open a therapeutic window for preventive immunomodulation and thereby reduce long-term morbidity. Clinically, these findings support careful follow-up after HSE, particularly during the first two months after infection, with a low threshold for NMDAR-IgG testing in CSF and serum when clinical deterioration suggests secondary NMDAR-E. Children and patients with signs of pronounced or prolonged CNS inflammation may represent particularly relevant groups for close monitoring, and future studies should assess whether HSV-specific signature antibodies can support early risk stratification after HSE.

Larger and ideally prospective surveillance and integrated immunological studies may further uncover additional, rarer virus-AIE associations, such as JEV-linked NMDAR-E, and refine our understanding of how infections shape human neuronal autoimmunity.

## Declaration of generative AI and AI-assisted technologies in the manuscript preparation process

During the preparation of this work, the authors used ChatGPT (OpenAI) as a supplementary tool to assist with phrasing. After using this tool, the authors reviewed and edited the content as needed and take full responsibility for the content of the published article.

## Funding

S.B. was supported by a BIH-MD scholarship from the Berlin Institute of Health at Charité and by a Deutschlandstipendium awarded through Charité – Universitätsmedizin Berlin. J.K. was supported by a Clinician Scientist fellowship from the Berlin Institute of Health at Charité. Furthermore, this work was supported by the German Research Council DFG (FOR3004, project number 415914819: KR5870/1-1) and by the Einstein Foundation Berlin (EZ-2023-751-2) to J.K.

## CRediT authorship contribution statement

**Sarah Bamberg:** Conceptualization, Data curation, Formal analysis, Investigation, Methodology, Visualization, Writing – original draft, Writing – review & editing. **Poul M. Schulte-Frankenfeld:** Data curation, Investigation, Visualization, Writing – review & editing. **Jakob Kreye:** Conceptualization, Formal analysis, Investigation, Methodology, Project administration, Supervision, Visualization, Writing – original draft, Writing – review & editing.

## Declaration of competing interest

The authors declare the following financial interests/personal relationships which may be considered as potential competing interests: Jakob Kreye has patent #European Patent Application No. 24187247.2: ‘Biomarkers for Herpes-simplex virus autoimmunity’ pending to Charité – Universitätsmedizin Berlin. If there are other authors, they declare that they have no known competing financial interests or personal relationships that could have appeared to influence the work reported in this paper.

## Data Availability

All reports from which data were extracted for this review are listed in Supplementary Table S2. Collected data used to generate figures and perform statistical tests will be available upon request to the corresponding author.
