# Supplementary material for: Associations of viral infections and antiviral vaccinations with anti-NMDAR and other forms of autoimmune encephalitis
Source: Brain Behav Immun Health. 2026 Jul 6;56:101301. doi: 10.1016/j.bbih.2026.101301 (PMC13380788; doi:10.1016/j.bbih.2026.101301)
Supplement: Multimedia component 1 [file mmc1.docx]

Supplementary data to the article

Associations of viral infections and antiviral vaccinations with anti-NMDAR and other forms of autoimmune encephalitis

Sarah Bamberg^a, b^, Poul M. Schulte-Frankenfeld^a, b^ and Jakob Kreye^a, b*^

^a^ *Charité – Universitätsmedizin Berlin, corporate member of Freie Universität Berlin and Humboldt-Universität zu Berlin, Department of Pediatric Neurology, Berlin, Germany*

^b^ *Berlin Institute of Health at Charité – Universitätsmedizin Berlin, Germany*

* Corresponding author at: Department of Pediatric Neurology, Charité – Universitätsmedizin Berlin, corporate member of Freie Universität Berlin, Humboldt-Universität zu Berlin, and Berlin Institute of Health, Augustenburger Platz 1, 13353 Berlin, Germany. E-Mail address: [jakob.kreye@charite.de](mailto:jakob.kreye@charite.de) (J. Kreye)

**List of Supplementary data**:

Supplementary Fig. S1.

Supplementary Fig. S2.

Supplementary Fig. S3.

Supplementary Table S1.

Supplementary Table S2.

Supplementary Table S3.

**
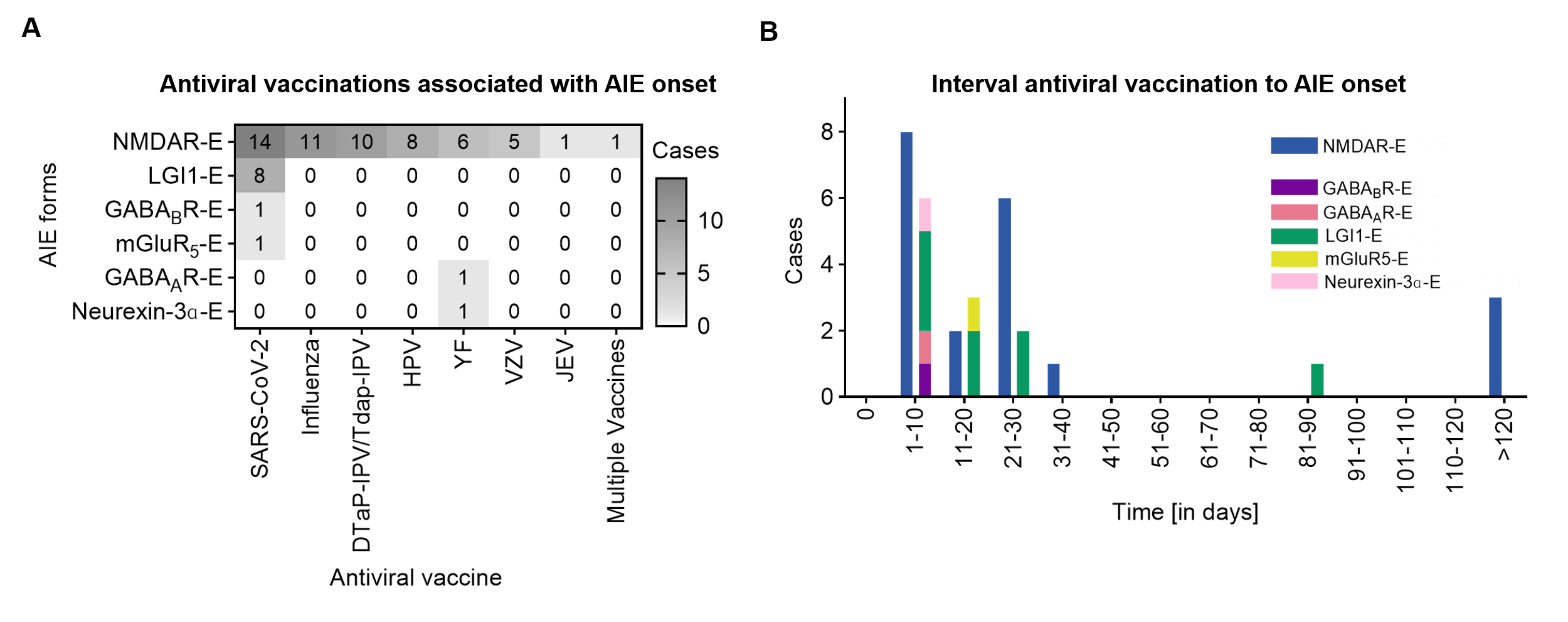
**

**Supplementary Fig. S1. Antiviral vaccinations associated with the onset of AIE. (A)** Heat map showing the absolute frequencies of antiviral vaccines that preceded the onset of NMDAR-E (*n* = 56) and non-NMDAR-E (*n* = 12). The NMDAR-E case associated with multiple vaccinations followed immunization against Hepatitis A and B, Rabies, Typhoid, and Yellow Fever. **(B)** Stacked bar plots show the time interval between antiviral vaccination and onset of NMDAR-E (*n* = 20) and non-NMDAR-E cases (*n* = 12).

DTaP-IPV/Tdap-IPV = Diphtheria, Tetanus, acellular Pertussis, and Inactivated Poliovirus Vaccine, HPV = Human Papillomavirus Vaccine, Influenza = Influenza Vaccine, JEV = Japanese Encephalitis Vaccine, SARS-CoV-2 = Severe Acute Respiratory Syndrome Coronavirus 2 Vaccine, VZV = Varicella-Zoster Virus Vaccine, YF = Yellow Fever Vaccine.

**
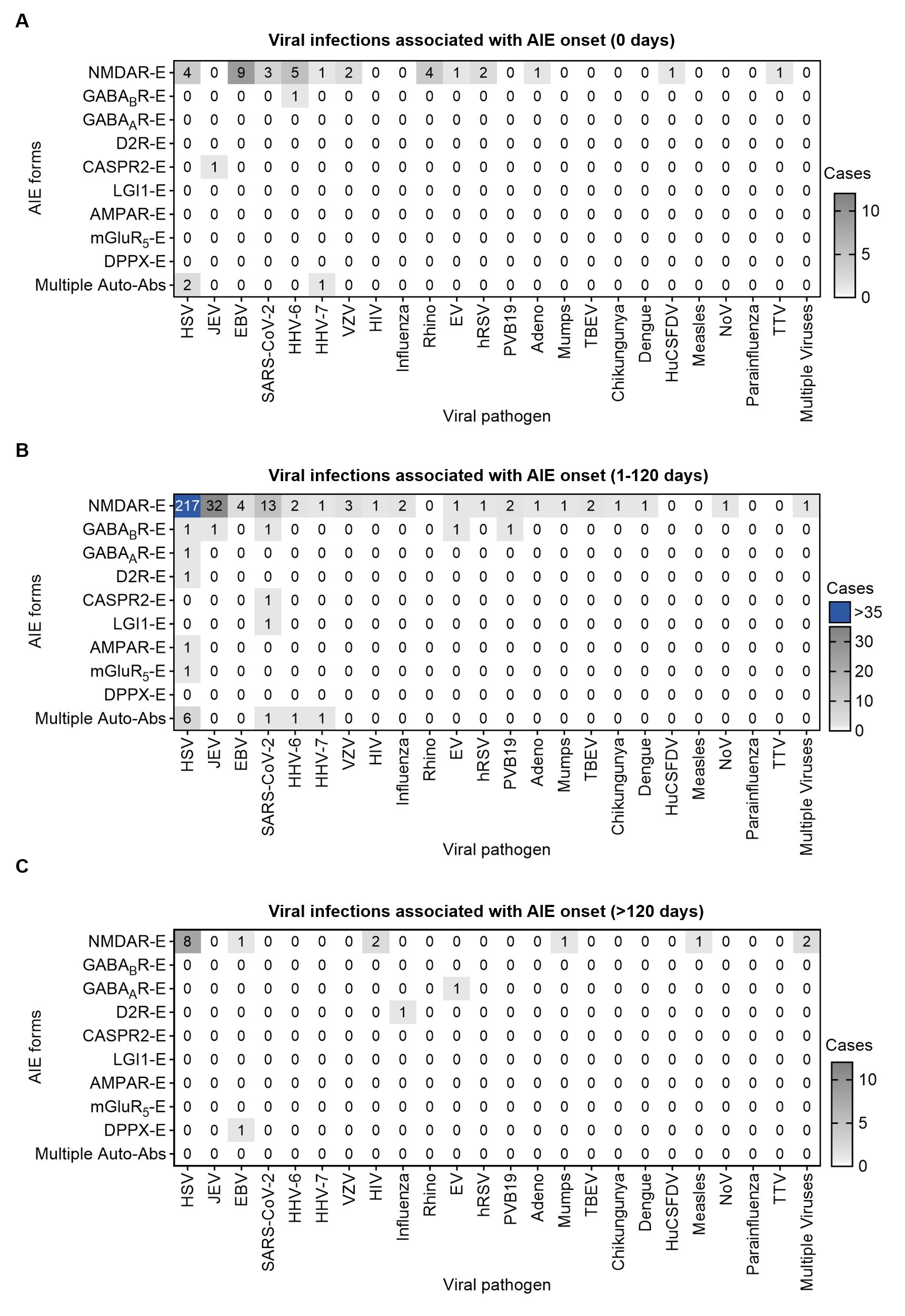
**

**Supplementary Fig. S2. Viral infections associated with the onset of AIE, stratified by time interval between infection and AIE onset. (A)** Heat map showing the absolute frequencies of detected viral species from infections reported in association with the onset of NMDAR-E (*n* = 34), non-NMDAR-E (*n* = 2), or cases with multiple autoantibodies (Multiple Auto-Abs; *n* = 3) at 0 days (concurrently detected infections, as defined in the Methods section). **(B)** Heat map showing the absolute frequencies of detected viral species from infections that occurred 1–120 days before onset of NMDAR-E (*n* = 287), non-NMDAR-E (*n* = 11), or cases with multiple autoantibodies (Multiple Auto-Abs; *n* = 9). **(C)** Heat map showing the absolute frequencies of detected viral species from infections that occurred more than 120 days before onset of NMDAR-E (*n* = 15), non-NMDAR-E (*n* = 3), or cases with multiple autoantibodies (Multiple Auto-Abs; *n* = 0).

Adeno = Adenovirus, Chikungunya = Chikungunya virus, Dengue = Dengue virus, EBV = Epstein-Barr virus, EV = Enterovirus, HIV = Human immunodeficiency virus, HHV-6 = Human herpesvirus 6, HHV-7 = Human herpesvirus 7, HuCSFDV = Human cerebrospinal fluid-associated densovirus, hRSV = Human respiratory syncytial virus, HSV = Herpes simplex virus, Influenza = Influenza virus, JEV = Japanese encephalitis virus, Measles = Measles virus, Mumps = Mumps virus, NoV = Norovirus, Parainfluenza = Human parainfluenza virus, PVB19 = Human parvovirus B19, Rhino = Rhinovirus, SARS-CoV-2 = Severe acute respiratory syndrome coronavirus 2, TBEV = Tick-borne encephalitis virus, TTV = Torque teno virus, VZV = Varicella-zoster virus.

**
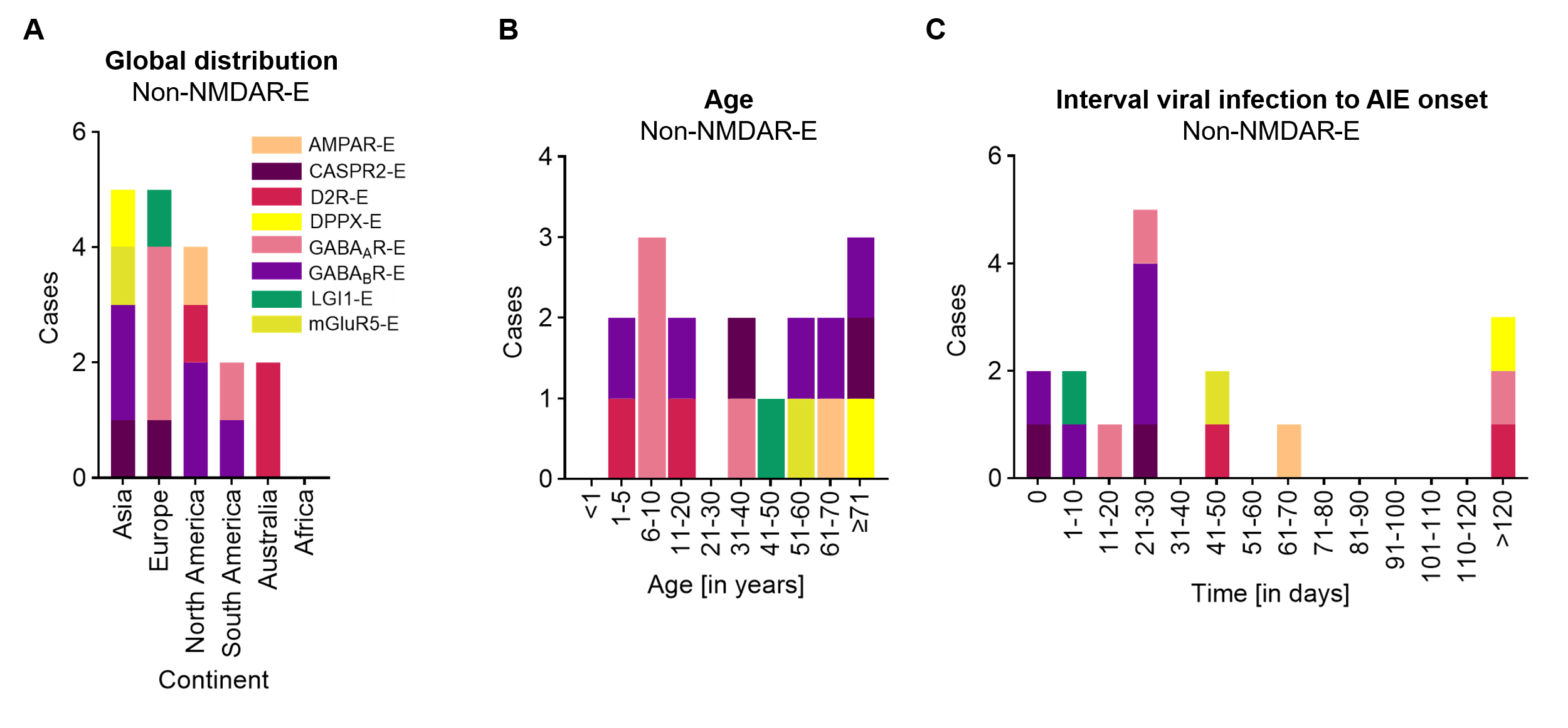
**

**Supplementary Fig. S3. Demographic features of viral infection-associated non-NMDAR-E cases. (A)** Stacked bar plots showing the global distribution by continent for viral infection-associated non-NMDAR-E cases (*n* = 18). South America includes cases from Central America. **(B)** Stacked bar plots indicating the age distribution for viral infection-associated non-NMDAR-E cases (*n* = 18). **(C)** Stacked bar plots showing the time interval between viral infection and onset of non-NMDAR-E (*n* = 16).

**Supplementary Table S1. PubMed search terms for AIE entities.**

| **AIE form** | **Search term** | **Year range** |
| --- | --- | --- |
| NMDAR-E | (NMDAR OR NMDA OR N-methyl-D-aspartate receptor) AND (encephalitis OR antibody OR antibodies) AND (virus OR viral OR vaccination OR vaccine) | 2007 – 2025 |
| AMPAR-E | (AMPA receptor OR α-Amino-3-hydroxy-5-methyl-4-isoxazolepropionic acid receptor OR AMPAR OR AMPA) AND (encephalitis OR antibody OR antibodies) AND (virus OR viral OR vaccination OR vaccine) | 2009 – 2025 |
| GABA_B_R-E | (GABA B receptor OR Gamma-aminobutyric acid B receptor OR GABAB OR Gamma-aminobutyric acid B) AND (encephalitis OR antibody OR antibodies) AND (virus OR viral OR vaccination OR vaccine) | 2009 – 2025 |
| LGI1-E | (LGI1 OR leucine rich glioma inactivated 1) AND (encephalitis OR antibody OR antibodies) AND (virus OR viral OR vaccination OR vaccine) | 2011 – 2025 |
| CASPR2-E | (CASPR2 OR Contactin Associated Protein-like 2 OR Contactin-associated protein 2) AND (encephalitis OR antibody OR antibodies) AND (virus OR viral OR vaccination OR vaccine) | 2011 – 2025 |
| D2R-E | (D2R OR Dopamine receptor D2 OR DRD2 OR D2 dopamine receptor OR Dopamine D2 receptor) AND (encephalitis OR antibody OR antibodies) AND (virus OR viral OR vaccination OR vaccine) | 2012 – 2025 |
| DPPX-E | (DPPX OR DPP6 OR Dipeptidyl aminopeptidase-related protein OR Dipeptidyl aminopeptidase-like protein 6) AND (encephalitis OR antibody OR antibodies) AND (virus OR viral OR vaccination OR vaccine) | 2013 – 2025 |
| GABA_A_R-E | (GABA A receptor OR Gamma-aminobutyric acid A receptor OR gabaa OR Gamma-aminobutyric acid A) AND (encephalitis OR antibody OR antibodies) AND (virus OR viral OR vaccination OR vaccine) | 2014 – 2025 |
| mGluR5-E | (metabotropic glutamate 5 receptor OR metabotropic glutamate receptor 5 OR mGluR5 OR mGlu5 OR GRM5) AND (encephalitis OR antibody OR antibodies) AND (virus OR viral OR vaccination OR vaccine) | 2014 – 2025 |
| Neurexin-3α-E | (Neurexin 3 alpha OR NRXN3 alpha OR Neurexin IIIα OR Neurexin-3α) AND (encephalitis OR antibody OR antibodies) AND (virus OR viral OR vaccination OR vaccine) | 2016 – 2025 |

**Supplementary Table S2. Included and excluded reports for all AIE forms.**

This table is provided as a standalone Excel file.

**Supplementary Table S3. Relative frequencies of HSV infections in viral encephalitis cohorts and CNS virus-associated NMDAR-E, by study.**

| **Viral encephalitis study** | | | **Age- and continent-matched subcohort of CNS virus-associated NMDAR-E** | | | ***P*-value from chi²** |
| --- | --- | --- | --- | --- | --- | --- |
|  | HSV cases (frequency) | Non-HSV cases (frequency) |  | HSV cases (frequency) | Non-HSV cases (frequency) |  |
| (George et al., 2014) (any age, North America) | 6698  (36.6%) | 11598  (63.4%) | this study (any age, North America) | 26  (66.7%) | 13  (33.3%) | *P* < 0.0001 |
| (Davison et al., 2003)  (any age, Europe) | 1419  (22.1%) | 4995  (77.9%) | this study  (any age, Europe) | 147  (91.9%) | 13  (8.1%) | *P* < 0.0001 |
| (Huppatz et al., 2009)  (any age, Australia) | 763  (61.4%) | 479  (38.6%) | this study  (any age, Australia) | 8  (100.0%) | 0  (0.0%) | *P* = 0.0253 |
| (Mailles et al., 2007)  (any age, Europe) | 337  (55.4%) | 271  (44.6%) | this study  (any age, Europe) | 147  (91.9%) | 13  (8.1%) | *P* < 0.0001 |
| (Koskiniemi et al., 2001)  (any age, Europe) | 48  (15.8%) | 256  (84.2%) | this study  (any age, Europe) | 147  (91.9%) | 13  (8.1%) | *P* < 0.0001 |
| (Mailles et al., 2022)  (any age, Europe) | 131  (50.8%) | 127  (49.2%) | this study  (any age, Europe) | 147  (91.9%) | 13  (8.1%) | *P* < 0.0001 |
| (Glaser et al., 2006)  (any age, North America) | 45  (30.6%) | 102  (69.4%) | this study (any age, North America) | 26  (66.7%) | 13  (33.3%) | *P* < 0.0001 |
| (Britton et al., 2020)  (age ≤ 17 yrs, Australia) | 17  (15.5%) | 93  (84.5%) | this study (age ≤ 17 yrs, Australia) | 5  (100.0%) | 0  (0.0%) | *P* < 0.0001 |
| (Sonneville et al., 2023)  (age ≥ 18 yrs, Europe) | 49  (48.5%) | 52  (51.5%) | this study (age ≥ 18 yrs, Europe) | 51  (85.0%) | 9  (15.0%) | *P* < 0.0001 |
| (Mailles et al., 2009)  (any age, Europe) | 55  (61.1%) | 35  (38.9%) | this study (any age, Europe) | 147  (91.9%) | 13  (8.1%) | *P* < 0.0001 |
| (Srey et al., 2002)  (any age, Asia) | 0  (0.0%) | 88  (100.0%) | this study  (any age, Asia) | 104  (70.7%) | 43  (29.3%) | *P* < 0.0001 |
| (Rantalaiho et al., 2001)  (age ≥ 18 yrs, Europe) | 37  (42.5%) | 50  (57.5%) | this study (age ≥ 18 yrs, Europe) | 51  (85.0%) | 9  (15.0%) | *P* < 0.0001 |
| (Kelly et al., 2013)  (any age, Europe) | 38  (49.4%) | 39  (50.6%) | this study (any age, Europe) | 147  (91.9%) | 13  (8.1%) | *P* < 0.0001 |
| (Lee et al., 2003)  (any age, Asia) | 45  (59.2%) | 31  (40.8%) | this study  (any age, Asia) | 104  (70.7%) | 43  (29.3%) | *P* = 0.0829 |
| (Palmas & Duke, 2023)  (age ≤ 17 yrs, Australia) | 5  (7.5%) | 62  (92.5%) | this study (age ≤ 17 yrs, Australia) | 5  (100.0%) | 0  (0.0%) | *P* < 0.0001 |
| (de Ory et al., 2013)  (any age, Europe) | 33  (53.2%) | 29  (46.8%) | this study (any age, Europe) | 147  (91.9%) | 13  (8.1%) | *P* < 0.0001 |
| (Granerod et al., 2010)  (any age, Europe) | 38  (65.5%) | 20  (34.5%) | this study (any age, Europe) | 147  (91.9%) | 13  (8.1%) | *P* < 0.0001 |
| (Olsen et al., 2015)  (any age, Asia) | 1  (2.8%) | 35  (97.2%) | this study  (any age, Asia) | 104  (70.7%) | 43  (29.3%) | *P* < 0.0001 |
| (Joshi et al., 2013)  (age ≥ 18 yrs, Asia) | 1  (3.2%) | 30  (96.8%) | this study  (age ≥ 18 yrs, Asia) | 20  (83.3%) | 4  (16.7%) | *P* < 0.0001 |
| (Quist-Paulsen et al., 2013)  (age ≥ 18 yrs, Europe) | 12  (54.5%) | 10  (45.5%) | this study (age ≥ 18 yrs, Europe) | 51  (85.0%) | 9  (15.0%) | *P* = 0.0038 |
| (Child et al., 2012)  (any age, Australia) | 4  (40.0%) | 6  (60.0%) | this study  (any age, Australia) | 8  (100.0%) | 0  (0.0%) | *P* = 0.0073 |
| (Frantzidou et al., 2008)  (any age, Europe) | 4  (50.0%) | 4  (50.0%) | this study (any age, Europe) | 147  (91.9%) | 13  (8.1%) | *P* = 0.0001 |

Please note that only 22 of the 23 studies are shown here, as there was no corresponding NMDAR-E comparison group for the African study by Sghaier *et al. (Sghaier et al., 2012).* For consistency reasons, chi² tests were applied for each statistical comparison despite limitations due to small sample sizes. HSV = Herpes Simplex Virus, NMDAR = N-methyl-D-aspartate receptor.

**Supplementary Material References**

1. Britton, P. N., Dale, R. C., Blyth, C. C., Clark, J. E., Crawford, N., Marshall, H., Elliott, E. J., Macartney, K., Booy, R., & Jones, C. A. (2020). Causes and Clinical Features of Childhood Encephalitis: A Multicenter, Prospective Cohort Study. *Clin Infect Dis*, *70*(12), 2517–2526. <https://doi.org/10.1093/cid/ciz685>
2. Child, N., Croxson, M. C., Rahnama, F., & Anderson, N. E. (2012). A retrospective review of acute encephalitis in adults in Auckland over a five-year period (2005-2009). *J Clin Neurosci*, *19*(11), 1483–1485. <https://doi.org/10.1016/j.jocn.2012.03.010>
3. Davison, K. L., Crowcroft, N. S., Ramsay, M. E., Brown, D. W., & Andrews, N. J. (2003). Viral encephalitis in England, 1989-1998: what did we miss? *Emerg Infect Dis*, *9*(2), 234–240. <https://doi.org/10.3201/eid0902.020218>
4. de Ory, F., Avellon, A., Echevarria, J. E., Sanchez-Seco, M. P., Trallero, G., Cabrerizo, M., Casas, I., Pozo, F., Fedele, G., Vicente, D., Pena, M. J., Moreno, A., Niubo, J., Rabella, N., Rubio, G., Perez-Ruiz, M., Rodriguez-Iglesias, M., Gimeno, C., Eiros, J. M.,…Vazquez-Moron, S. (2013). Viral infections of the central nervous system in Spain: a prospective study. *J Med Virol*, *85*(3), 554–562. <https://doi.org/10.1002/jmv.23470>
5. Frantzidou, F., Kamaria, F., Dumaidi, K., Skoura, L., Antoniadis, A., & Papa, A. (2008). Aseptic meningitis and encephalitis because of herpesviruses and enteroviruses in an immunocompetent adult population. *Eur J Neurol*, *15*(9), 995–997. <https://doi.org/10.1111/j.1468-1331.2008.02233.x>
6. George, B. P., Schneider, E. B., & Venkatesan, A. (2014). Encephalitis hospitalization rates and inpatient mortality in the United States, 2000-2010. *PLoS One*, *9*(9), e104169. <https://doi.org/10.1371/journal.pone.0104169>
7. Glaser, C. A., Honarmand, S., Anderson, L. J., Schnurr, D. P., Forghani, B., Cossen, C. K., Schuster, F. L., Christie, L. J., & Tureen, J. H. (2006). Beyond viruses: clinical profiles and etiologies associated with encephalitis. *Clin Infect Dis*, *43*(12), 1565–1577. <https://doi.org/10.1086/509330>
8. Granerod, J., Ambrose, H. E., Davies, N. W., Clewley, J. P., Walsh, A. L., Morgan, D., Cunningham, R., Zuckerman, M., Mutton, K. J., Solomon, T., Ward, K. N., Lunn, M. P., Irani, S. R., Vincent, A., Brown, D. W., Crowcroft, N. S., & Group, U. K. H. P. A. A. o. E. S. (2010). Causes of encephalitis and differences in their clinical presentations in England: a multicentre, population-based prospective study. *Lancet Infect Dis*, *10*(12), 835–844. <https://doi.org/10.1016/S1473-3099(10)70222-X>
9. Huppatz, C., Durrheim, D. N., Levi, C., Dalton, C., Williams, D., Clements, M. S., & Kelly, P. M. (2009). Etiology of encephalitis in Australia, 1990-2007. *Emerg Infect Dis*, *15*(9), 1359–1365. <https://doi.org/10.3201/eid1509.081540>
10. Joshi, R., Mishra, P. K., Joshi, D., Santhosh, S. R., Parida, M. M., Desikan, P., Gangane, N., Kalantri, S. P., Reingold, A., & Colford, J. M., Jr. (2013). Clinical presentation, etiology, and survival in adult acute encephalitis syndrome in rural Central India. *Clin Neurol Neurosurg*, *115*(9), 1753–1761. <https://doi.org/10.1016/j.clineuro.2013.04.008>
11. Kelly, T. A., O'Lorcain, P., Moran, J., Garvey, P., McKeown, P., Connell, J., & Cotter, S. (2013). Underreporting of viral encephalitis and viral meningitis, Ireland, 2005-2008. *Emerg Infect Dis*, *19*(9), 1428–1436. <https://doi.org/10.3201/eid1909.130201>
12. Koskiniemi, M., Rantalaiho, T., Piiparinen, H., von Bonsdorff, C. H., Farkkila, M., Jarvinen, A., Kinnunen, E., Koskiniemi, S., Mannonen, L., Muttilainen, M., Linnavuori, K., Porras, J., Puolakkainen, M., Raiha, K., Salonen, E. M., Ukkonen, P., Vaheri, A., Valtonen, V., & Study, G. (2001). Infections of the central nervous system of suspected viral origin: a collaborative study from Finland. *J Neurovirol*, *7*(5), 400–408. <https://doi.org/10.1080/135502801753170255>
13. Lee, T. C., Tsai, C. P., Yuan, C. L., Wei, C. Y., Tsao, W. L., Lee, R. J., Cheih, S. Y., Huang, I. T., & Chen, K. T. (2003). Encephalitis in Taiwan: a prospective hospital-based study. *Jpn J Infect Dis*, *56*(5-6), 193–199.
14. Mailles, A., Argemi, X., Biron, C., Fillatre, P., De Broucker, T., Buzele, R., Gagneux-Brunon, A., Gueit, I., Henry, C., Patrat-Delon, S., Makinson, A., Piet, E., Wille, H., Vareil, M. O., Epaulard, O., Martinot, M., Tattevin, P., Stahl, J. P., scientific, c., & investigators. (2022). Changing profile of encephalitis: Results of a 4-year study in France. *Infect Dis Now*, *52*(1), 1–6. <https://doi.org/10.1016/j.idnow.2021.11.007>
15. Mailles, A., Stahl, J. P., Steering, C., & Investigators, G. (2009). Infectious encephalitis in france in 2007: a national prospective study. *Clin Infect Dis*, *49*(12), 1838–1847. <https://doi.org/10.1086/648419>
16. Mailles, A., Vaillant, V., & Stahl, J. P. (2007). [Infectious encephalitis in France from 2000 to 2002: the hospital database is a valuable but limited source of information for epidemiological studies]. *Med Mal Infect*, *37*(2), 95–102. <https://doi.org/10.1016/j.medmal.2006.11.001> (Encephalites infectieuses: donnees et limites du PMSI pour l'etude epidemiologique, France metropolitaine 2000-2002.)
17. Olsen, S. J., Campbell, A. P., Supawat, K., Liamsuwan, S., Chotpitayasunondh, T., Laptikulthum, S., Viriyavejakul, A., Tantirittisak, T., Tunlayadechanont, S., Visudtibhan, A., Vasiknanonte, P., Janjindamai, S., Boonluksiri, P., Rajborirug, K., Watanaveeradej, V., Khetsuriani, N., Dowell, S. F., & Thailand Encephalitis Surveillance, T. (2015). Infectious causes of encephalitis and meningoencephalitis in Thailand, 2003-2005. *Emerg Infect Dis*, *21*(2), 280–289. <https://doi.org/10.3201/eid2102.140291>
18. Palmas, G., & Duke, T. (2023). Severe encephalitis: aetiology, management and outcomes over 10 years in a paediatric intensive care unit. *Arch Dis Child*, *108*(11), 922–928. <https://doi.org/10.1136/archdischild-2023-325305>
19. Quist-Paulsen, E., Kran, A. M., Dunlop, O., Wilson, J., & Ormaasen, V. (2013). Infectious encephalitis: a description of a Norwegian cohort. *Scand J Infect Dis*, *45*(3), 179–185. <https://doi.org/10.3109/00365548.2012.719634>
20. Rantalaiho, T., Farkkila, M., Vaheri, A., & Koskiniemi, M. (2001). Acute encephalitis from 1967 to 1991. *J Neurol Sci*, *184*(2), 169–177. <https://doi.org/10.1016/s0022-510x(01)00441-5>
21. Sghaier, W., Bahri, O., Kedous, E., Fazaa, O., Rezig, D., Touzi, H., Ben Yahia, A., Meddeb, Z., & Triki, H. (2012). [Retrospective study of viral causes of central nervous system infections in Tunisia (2003-2009)]. *Med Sante Trop*, *22*(4), 373–378. <https://doi.org/10.1684/mst.2012.0080> (Etude retrospective des etiologies virales des infections neuromeningees en Tunisie (2003-2009).)
22. Sonneville, R., de Montmollin, E., Contou, D., Ferrer, R., Gurjar, M., Klouche, K., Sarton, B., Demeret, S., Bailly, P., da Silva, D., Escudier, E., Le Guennec, L., Chabanne, R., Argaud, L., Ben Hadj Salem, O., Thyrault, M., Frerou, A., Louis, G., De Pascale, G.,…Group, E. I. S. (2023). Clinical features, etiologies, and outcomes in adult patients with meningoencephalitis requiring intensive care (EURECA): an international prospective multicenter cohort study. *Intensive Care Med*, *49*(5), 517–529. <https://doi.org/10.1007/s00134-023-07032-9>
23. Srey, V. H., Sadones, H., Ong, S., Mam, M., Yim, C., Sor, S., Grosjean, P., & Reynes, J. M. (2002). Etiology of encephalitis syndrome among hospitalized children and adults in Takeo, Cambodia, 1999-2000. *Am J Trop Med Hyg*, *66*(2), 200–207. <https://doi.org/10.4269/ajtmh.2002.66.200>
